# Multi-electron transfer enabled by topotactic reaction in magnetite

**DOI:** 10.1038/s41467-019-09528-9

**Published:** 2019-04-29

**Authors:** Wei Zhang, Yan Li, Lijun Wu, Yandong Duan, Kim Kisslinger, Chunlin Chen, David C. Bock, Feng Pan, Yimei Zhu, Amy C. Marschilok, Esther S. Takeuchi, Kenneth J. Takeuchi, Feng Wang

**Affiliations:** 1 Sustainable Energy Technologies Department, Brookhaven National Laboratory, Upton, NY 11973 USA; 2 American Physical Society, Ridge, NY 11961 USA; 3 Condensed Matter Physics and Materials Science Department, Brookhaven National Laboratory, Upton, NY 11973 USA; 4 School of Advanced Materials, Peking University, Shenzhen Graduate School, Shenzhen, 518055 China; 5 Center for Functional Nanomaterials, Brookhaven National Laboratory, Upton, NY 11973 USA; 6 Shenyang National Laboratory for Materials Science, Institute of Metal Research, Chinese Academy of Sciences, Shenyang, 110016 China; 7 Energy Sciences Directorate, Brookhaven National Laboratory, Upton, NY 11973 USA; 8 Department of Materials Science and Engineering, Stony Brook University, Stony Brook, NY 11794 USA; 9 Department of Chemistry, Stony Brook University, Stony Brook, NY 11794 USA

**Keywords:** Batteries, Batteries

## Abstract

A bottleneck for the large-scale application of today’s batteries is low lithium storage capacity, largely due to the use of intercalation-type electrodes that allow one or less electron transfer per redox center. An appealing alternative is multi-electron transfer electrodes, offering excess capacity, which, however, involves conversion reaction; according to conventional wisdom, the host would collapse during the process, causing cycling instability. Here, we report real-time observation of topotactic reaction throughout the multi-electron transfer process in magnetite, unveiled by in situ single-crystal crystallography with corroboration of first principles calculations. Contradicting the traditional belief of causing structural breakdown, conversion in magnetite resembles an intercalation process—proceeding via topotactic reaction with the cubic close packed oxygen-anion framework retained. The findings from this study, with unique insights into enabling  multi-electron transfer via topotactic reaction, and its implications to the cyclability and rate capability, shed light on designing viable multi-electron transfer electrodes for high energy batteries.

## Abstract (web summary)

In contrast to the conventional wisdom on conversion-driven structural collapse of the host, this work shows that lithium conversion in magnetite resembles the intercalation process, going via topotactic reactions, thereby enabling multi-electron transfer and high reversible capacity.

## Introduction

Intercalation-type electrode materials are commonly employed in commercial lithium-ion batteries (LIBs), as they accommodate Li via topotactic transformation, conducive to small structural change over cycling^1^. Unfortunately, their capacity is low, limited by one or less electron transfer per redox center, which has been a bottleneck for the large-scale applications^2,3^. In contrast, multi-electron transfer (MET) electrode materials, such as transition metal (TM) compounds (TM_a_X_b_, where TM: Fe, Co, Ni, …; X: O, F, S, …), hold great promise for use in next-generation LIBs due to their extremely high capacity^4–8^. Nonetheless, Li conversion is involved during MET reaction, and according to the traditional view, the lattice  structure of the host will break down upon lithiation, i.e., from the initial TM_a_X_b_ into the nano-sized TM and Li_n_X (where n is the formal oxidation state of X), and therefore degrades over cycling^9–12^.

One exception is inverse-spinel structured magnetite (Fe_3_O_4_;  space group: Fd$\bar 3$m)^13,14^, wherein high cyclability has been demonstrated^20–22^^,^^23^. And due to its high capacity, low cost, and natural abundance, Fe_3_O_4_ is of great interest for battery application^12,15–19^. The recent mechanistic studies on electrochemical reaction in Fe_3_O_4_ provided insights into the structure-determined ionic transport and high cyclability in this material^12,16,24–26^. Particularly, a combination of synchrotron X-ray and electron microscopy measurements revealed preferential orientation of the formed  Fe nanoparticles^12^. A similar phenomenon has also been observed in Co_3_O_4_ spinel^27^ and FeF_2_^10,11^, suggesting a crystallographic correlation between the parent reactant and converted phases despite their abrupt structural difference. In addition, the formation of metallic nanoparticles has been shown to improve the electronic conductivity and reversibility of the electrodes^9,10,28^, but it is still unclear how those metallic nanoparticles initially nucleate and grow from the parent or intermediate phases, and consequently, impact the conversion process during lithiation. Since batteries generally operate under an overpotential, little is known about the dynamic process of structural evolution and the involved formation of metallic nanoparticles under non-equilibrium conditions.

Traditional X-ray diffraction based in situ techniques have been widely used for obtaining information on structural changes over large areas of the electrode materials, which, however, cannot catch the progression of the reaction front, namely, where and how the reaction is initialized and how the involved phases evolve and structurally correlate with each other throughout the reaction process^29^. In situ transmission electron microscopy (TEM) approaches, such as electron diffraction (ED), have been proven to be powerful in tracking the local structural evolution in the selected areas or individual particles^29–33^. In the particular systems that undergo MET reaction, multi-phase transformations occur within nano-sized domains, which have often been complicated by the  use of polycrystalline materials, since the random orientation of constituent crystallites obscures the crystallographic relationship between the involved intermediates as they develop^30^. Herein, single-crystal samples are employed for in situ ED studies of the MET reaction in Fe_3_O_4_, allowing us to track the associated crystallographic evolution during lithiation. The crystallographic correlation between all of the involved phases during intercalation and conversion processes are revealed, indicating co-operative migration of Li and Fe ions within the O-anion cubic close-packed (ccp) framework. Contradicting the conventional belief of causing structural breakdown, conversion in Fe_3_O_4_ resembles the intercalation process, going via topotactic reaction with the ccp oxygen-anion framework retained, thereby enabling multi-electron transfer in this material. The experimental observation is further coupled to first principles calculations, allowing identification of ionic transport pathway, and  making direct correlation to the cyclability and rate capability in Fe_3_O_4_.

## Results

### Real time observation of topotactic reaction in single-crystal Fe_3_O_4_

**Fig. 1:**
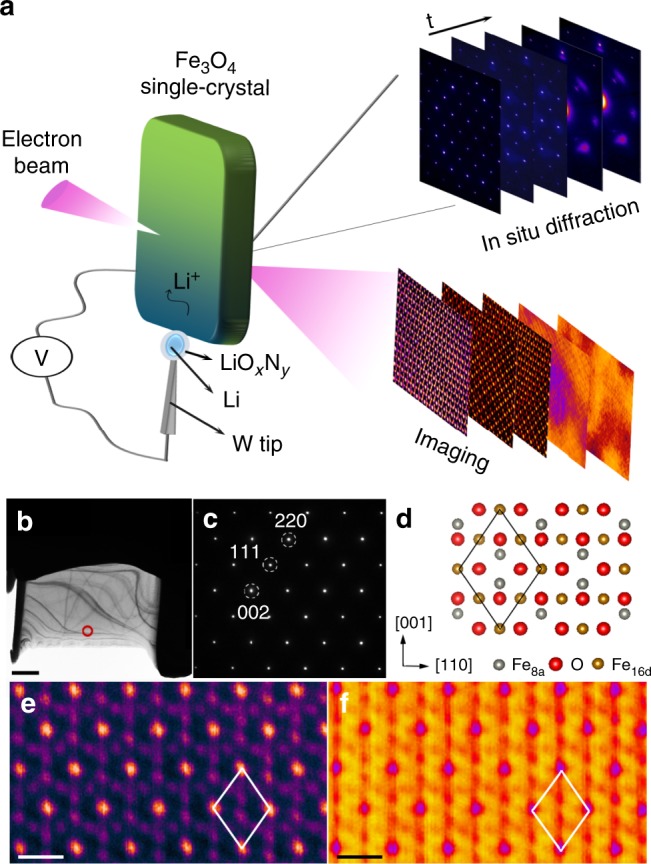
Experimental setup for tracking electrochemical reaction in a single crystal of Fe_3_O_4_. **a** Schematic illustration of the experimental design in this study that allows probing of structural evolution at the reaction front, via a combination of time-resolved in situ electron diffraction and spatially-resolved atomic imaging (i.e., providing “snapshots” at certain lithiation states). An in situ electrochemical cell specialized for crystallographic studies is designed, with a Fe_3_O_4_ single-crystal as an active electrode, Li metal as a counter electrode and natively formed LiO_*x*_N_*y*_ as a solid electrolyte. The individual components of the cell are connected using a piezo-driven biasing probe built into the TEM–scanning tunneling microscopy sample stage. **b**, **c** Bright-field TEM image of the Fe_3_O_4_ sample, with a large thinned area (brighter) for this study, and the corresponding electron diffraction pattern from the selected area (as labeled by a red circle in (**b**)), indicating its orientation along the [1$\bar1$0] direction. Scale bar: 1 µm. **d** Structure model of the atomic arrangement viewed along the [1$\bar1$0] direction, showing the occupancy of Fe at 8a/16d sites and O at 32e sites in the inverse spinel structure (space group: Fd$\bar3$m). **e**, **f** HAADF and ABF images taken simultaneously from the edge of the slice in (**b**), as labeled by the red circle. Fe atoms can be visualized in the HAADF image, while the O and Fe become distinguishable in the ABF image. Scale bars: 0.5 nm

Figure 1a shows the experimental  setup that was used to perform time-resolved in situ ED and spatially-resolved atomic imaging at the reaction front. A thin slice sample was cut from one large single crystal along the zone axis [1$\bar 1$0], as shown in Fig. 1b (see also details in the Supplementary Methods and Supplementary Fig. 1 in the Supplementary Information). Compared to polycrystalline materials with random orientations, such type of sample is advantageous in interrogating the crystallographic correlation between the pristine and all of the lithiated phases by tracking changes in the highly relevant planes, such as {002}, {111} and {220}, from the corresponding diffraction spots (Fig. 1c). Complementary to in situ ED experiments on crystallographic evolution in the bulk, atomic imaging enabled by aberration-corrected high angle annular-dark-field (HAADF) and annular-bright-field (ABF) techniques, was also used for identifying atomic arrangements of anions and cations, locally at the pristine and partially lithiated states. Occupancies of Fe cations at the tetrahedral sites (8a) and octahedral sites (16d), and O anions at the 32e sites in Fe_3_O_4_, were unambiguously determined by HAADF and ABF imaging (Fig. 1d–f).

**Fig. 2:**
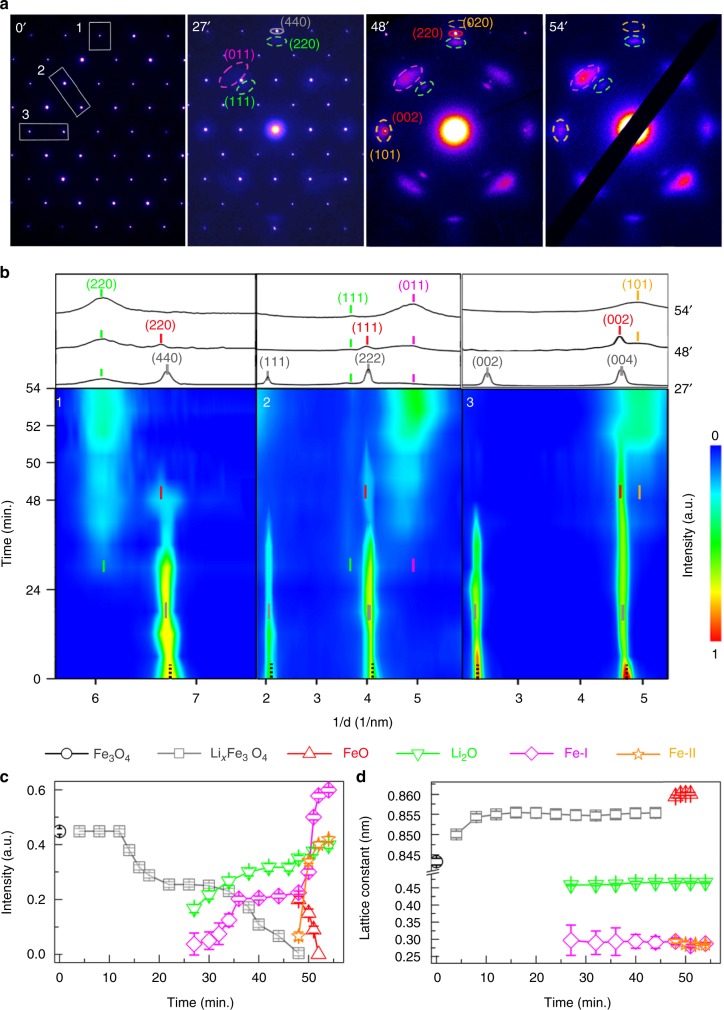
Topotactic reaction pathways during multi-electron transfer in Fe_3_O_4_ revealed by in situ electron diffraction crystallography. **a** Represenative diffraction patterns recorded at 0′, 27′, 48′, 54′ (as labeled), respectively. The reflection spots associated with the initial Fe_3_O_4_, intermediate Li_*x*_Fe_3_O_4_ (being marked by a gray circle), FeO (red), and final phases of Li_2_O (green), Fe-I (purple) and Fe-II (orange) indicate the crystallographic orientation of the Fe_3_O_4_/Li_*x*_Fe_3_O_4_, FeO, Li_2_O, Fe-I and Fe-II phases, along the [1$\bar1$0], [1$\bar1$0], [1$\bar1$0], [100] and [10$\bar1$] directions, respectively, with well-defined correlation. See also details further elaborated by the movie (Supplementary Movie 1 on the whole process), and indexing of all the diffraction spots (Supplementary Fig. 2) in the Supplementary Information. **b** Intensity maps of the diffraction peaks (bottom), along 3 different crystallograhic directions as labeled by the boxes in (**a**) at 0′, and the corresponding intensity profiles at some of the intermediate/final states (top; at 27′, 48′, 54′ as labeled). **c** Integrated intensities of (111)_Fe3O4_, (111)_Li*x*Fe3O4_, (111)_FeO_, (220)_Li2O_, (011)_Fe-I_ and (101)_Fe-II_ as a function of lithiation time. **d** Evolution of lattice constant of the involved intermediate phases. The evolution of peak intensity and lattice constant demonstrates a multiple-stage phase transformation, from the intitial Fe_3_O_4_ to intermediates Li_*x*_Fe_3_O_4_, FeO, and final phases, Fe-I, Fe-II, Li_2_O. The lattice constant of FeO is doubled for comparison with other ones. The error bars represent standard deviations of the fitting of intensity profiles (using a Gaussian function)

Figure 2 shows the main results from time-resolved ED measurements conducted on the Fe_3_O_4_ single-crystal during lithiation. Representative patterns recorded at the pristine, intermediate/final states (Fig. 2a), clearly show the crystallographic correlation between all of the involved phases. During initial lithiation, all the reflection spots of the Fe_3_O_4_ (Fig. 2a at 0′) turned into the spots of intermediate Li_*x*_Fe_3_O_4_ (Fig. 2a at 27′). Spots associated with Li_2_O and metallic Fe projected along the [100] direction (Case-I from hereafter), were also detected by 27′ although they are diffusive and weak (Fig. 2a), and importantly, they all appeared at certain positions adjacent to the reflection spots of Li_*x*_Fe_3_O_4_, indicating certain crystallographic correlation between those phases. By 48′, all of the spots associated with Li_*x*_Fe_3_O_4_ turned into the ones of FeO, and in the meanwhile those ones associated with Fe-I and Li_2_O phases became more intense (Fig. 2a). Additional spots associated with {101}_Fe_ group were also identified, but they don’t belong to Fe-I, and have never been reported in previous work^12,30^, so-called Case-II from hereafter. Together with the spots belonging to the group of {020}_Fe-II_, the Fe-II phase was determined to project along the [$10\bar 1$] zone axis. At the fully lithiated state (54′), the diffraction spots associated with FeO were completely absent in the pattern, indicating the full conversion into Li_2_O and Fe phases (Fig. 2a).

 Crystallographic evolution as a function of time is shown by the false-colored intensity map related to several specific planes, and the corresponding intensity profiles (Fig. 2b). Further quantitative analysis was also made to the evolution in the intensities of diffraction spots, and related lattice changes of all the involved phases (as given in Fig. 2c and d), which highlights two different sequences in the phase transformation. In Sequence 1, the phase evolution followed Fe_3_O_4_ → Li_*x*_Fe_3_O_4_ → Li_2_O and Fe-I. During this process, lithiation proceeded continuously until the 16th minute, during which all the peaks associated with pristine Fe_3_O_4_ shifted to lower angles, indicating an increase in the lattice constant and the related, gradual transformation from Fe_3_O_4_ to Li_*x*_Fe_3_O_4_ (Fig. 2d). By the 27th minute, some portion of Li_*x*_Fe_3_O_4_ started to be converted into Li_2_O and Fe-I, as shown by the appearance of (220)_Li2O_ peak along the line scan 1, and (111)_Li2O_ and (011)_Fe-I_ peaks along the line scan 2 (Fig. 2a, b and Supplementary Fig. 2a). At this stage, the spots related to the FeO phase were not detected anymore (Fig. 2b).

A second sequence of phase evolution was also found (Sequence 2): Fe_3_O_4_ → Li_*x*_Fe_3_O_4_ → FeO →Li_2_O, Fe-I and Fe-II. Starting from the 48th minute, the Li_*x*_Fe_3_O_4_ phase was converted into FeO first, as shown by the evolution of diffraction peaks (Fig. 2b). Specifically, the peaks of (111)_Li*x*Fe3O4_ in the line scan 2 and (002)_Li*x*Fe3O4_ in the line scan 3 disappeared, whereas the peaks of (440)_Li*x*Fe3O4_, (222)_Li*x*Fe3O4_ and (004)_Li*x*Fe3O4_ further shifted to the left and became (220)_FeO_, (111)_FeO_ and (002)_FeO_, respectively (Fig. 2b and Supplementary Fig. 2b). The FeO phase was further transformed to Li_2_O and Fe-I/Fe-II, as indicated by the substantial increase in the intensities of (220)_Li2O_, (011)_Fe-I_ and (101)_Fe-II_ (Fig. 2c). It should be noted the formation of Fe-II occurred at a stage much later than that of Fe-I, and that the intensities of peaks related to Fe-II were significantly weaker than those of Fe-I (Fig. 2c), suggesting that Fe-I is the dominant type of metallic Fe formed during the conversion reaction.

The direct transformation from Li_*x*_Fe_3_O_4_ to the final Fe phase in the Sequence 1 (i.e., without going through FeO) is basically the same as that observed by in situ ED measurements on agglomerate Fe_3_O_4_ particles^30^. However, in the present work, the use of single crystal enabled, for the first time, the identification of the second different sequence of phase transformation in local regions, by first forming the rocksalt-like phase Li_*x*_Fe_3_O_4_ (0 < *x* < 2), followed by the formation of the highly structurally-integrated FeO and Li_2_O domains as evidenced by a high-resolution TEM image in Supplementary Figure 3, and finally the Li_2_O and Fe phases^12^. Such local information may have been obfuscated by the agglomerate behavior and reaction inhomogeneity in a polycrystalline powder sample^30^.

All the above observations indicate a topotactic relationship between the pristine Fe_3_O_4_ and the intermediate/final phases, Li_*x*_Fe_3_O_4_, FeO, Li_2_O, Fe-I, and Fe-II, as explicitly given below:

$$\begin{array}{l}\left[ {1\bar 10} \right]_{{\mathrm{Fe3O4}}}//\left[ {1\bar 10} \right]_{{\mathrm{FeO}}}//\left[ {1\bar 10} \right]_{{\mathrm{Li2O}}},\left( {440} \right)_{{\mathrm{Fe3O4}}}//\left( {220} \right)_{{\mathrm{FeO}}}\\ //\left( {220} \right)_{{\mathrm{Li2O}}},\left( {004} \right)_{{\mathrm{Fe3O4}}}//\left( {002} \right)_{{\mathrm{FeO}}}//\left( {002} \right)_{{\mathrm{Li2O}}};\\ \left[ {1\bar 10} \right]_{{\mathrm{FeO}}} //\left[ {100} \right]_{{\mathrm{Fe}} {\hbox{-}} {\mathrm{I}}},\left( {220} \right)_{{\mathrm{FeO}}}//\left( {010} \right)_{{\mathrm{Fe}} {\hbox{-}} {\mathrm{I}}}\left( {002} \right)_{{\mathrm{FeO}}}//\left( {001} \right)_{{\mathrm{Fe}} {\hbox{-}} {\mathrm{I}}};\\ \left[ {1\bar 10} \right]_{{\mathrm{FeO}}}//\left[ {10\bar 1} \right]_{{\mathrm{Fe}} {\hbox{-}} {\mathrm{II}}},\left( {220} \right)_{{\mathrm{FeO}}}//\left( {010} \right)_{{\mathrm{Fe}} {\hbox{-}} {\mathrm{II}}}\left( {002} \right)_{{\mathrm{FeO}}}//\left( {101} \right)_{{\mathrm{Fe}} {\hbox{-}} {\mathrm{II}}}.\end{array}$$

The observation of the crystallographic correlation between Fe_3_O_4_, Li_*x*_Fe_3_O_4_, FeO, Li_2_O, and Fe-I is consistent with previous ex situ results obtained using coin cells^12^. But through in situ observation on the single crystal, the dynamic process of crystallographic transformation has been revealed in detail. Furthermore, by tracking the formation of Li_2_O and Fe-II phases (not reported previously) along with other involved phases (Li_*x*_Fe_3_O_4_, FeO), and their crystallographic orientation, we were able to unambiguously determine the topotactic pathway throughout the entire process, during which all of the involved phases share the same ccp O-anion framework (as further discussed below). Such a topotactic pathway has also been observed on additional single-crystal samples (see Supplementary Fig. 4 and Supplementary Note 1). The process of phase transformation, two sequences of phase evolution, the crystallographic correlation between different phases and the formation of Fe-II phase are all the same as that in Fig. 2. Therefore, in addition to offering more details about structural evolution than what was reported in previous work^12^, the in situ studies provided new insights into the crystallographic correlation between them, which can only be achievable using the newly developed methodology in the present work.

### Spatial correlation between intermediates

**Fig. 3:**
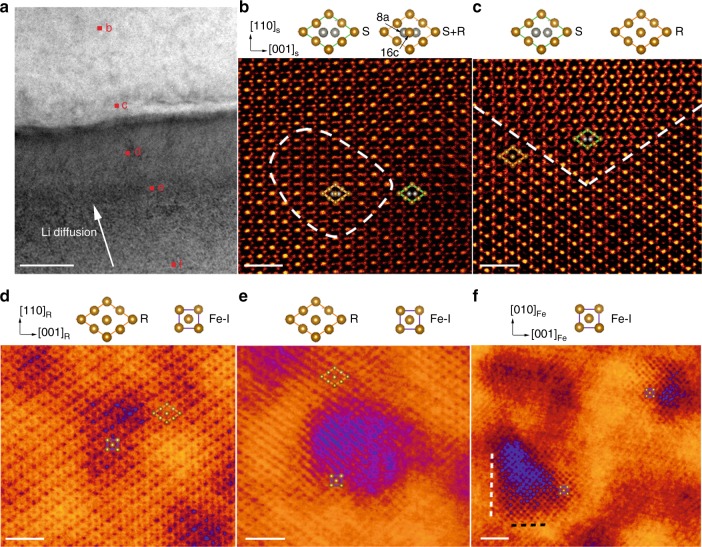
Spatial correlation among the pristine, intermediate and final phases in a partially lithiated Fe_3_O_4_ single-crystal by atomic imaging. **a** Bright field TEM image from a large area of the single crystal that was partially lithiated to a series of different states, shown by diffraction contrast. The direction of Li diffusion is indicated by the white arrow. Scale bar: 100 nm. **b**–**f** HAADF images taken at the local regions as labeled by the letters of b, c, d, e, f respectively, in (**a**). Scale bar: 1 nm. The spinel (S) and rocksalt-like Li_*x*_Fe_3_O_4_ (R) phases are both projected along the [1$\bar1$0] direction. Dashed lines in (**b**) and (**c**) label the boundaries between S and the mixture of S and R, and that between S and R, respectively. The Fe-I nanograins are projected along the [100] direction, as marked by purple in (**e**) and (**f**). The interfaces between R and Fe-I, i.e., {001}_Fe-I_ and {010}_Fe-I_, are marked by white and black dashed lines in (**f**), respectively. The atomic arrangements of Fe in S, R and Fe-I are shown on the top of each HAADF image

Complementary to in situ ED on crystallographic evolution in the bulk crystals, HAADF images were taken from a partially-lithiated Fe_3_O_4_ single-crystal (as given in Fig. 3). A series of atomic images were taken from local regions, showing different nucleation and growth states of the intermediate phases: from the initial spinel Fe_3_O_4_ (labeled as “S”) to rocksalt-like Li_*x*_Fe_3_O_4_ phase (labeled as “R”), and then to Fe (see also Supplementary Fig. 5). The Fe_3_O_4_, Li_*x*_Fe_3_O_4_, and Fe phases located at different regions, due to their different diffraction contrast (as shown in Fig. 3a), were well distinguished from each other and further identified by the fast Fourier transform (FFT) patterns of the HAADF images (shown in Supplementary Fig. 5). In some regions (e.g., b), small grains of Li_*x*_Fe_3_O_4_ were found within the matrix of the spinel phase (Fig. 3b). At the early lithiated state, the inserted Li ions first occupy the empty octahedral 16c sites in Fe_3_O_4_,  and so force the adjacent Fe ions at 8a to move to the neighboring 16c sites, leading to the formation of rocksalt-like Li_*x*_Fe_3_O_4_ structure^34^. Due to the projected view along the [$1\bar 10$] direction, Fe ions at the tetrahedral 8a sites of Fe_3_O_4_ and those at the octahedral 16c site of Li_*x*_Fe_3_O_4_ appear nearly overlapped in the HAADF images (Fig. 3b). In the deeper lithiated region (Fig. 3c), Li_*x*_Fe_3_O_4_ became populated, forming a sharp interface of {111} planes between Fe_3_O_4_ and Li_*x*_Fe_3_O_4_ (as marked by the dashed line). In region d (of an even deeper lithiated state), Li_*x*_Fe_3_O_4_ were partially converted to Fe-I, in the form of nanograins, giving rise to a different contrast in Fig. 3d (in purple). At regions of even deeper lithiation, the Fe-I nanograins were still embedded inside Li_*x*_Fe_3_O_4_, but grew out in the region (Fig. 3e). The Fe-I nanograins were projected along the [100] direction, exactly the same as the projection detected by ED (Fig. 2). In the region f at the full lithiated state, only multiple Fe-I nanograins, aligned along the same orientation, were observed (Fig. 3f). The interfaces between Fe-I nanograins and ccp framework were marked by white and black dashed lines in Fig. 3f, corresponding to {001}_Fe-I_ // {001}_ccp_ and {010}_Fe-I_ // {110}_ccp_, respectively. Moreover, structural transformation from FeO to Fe-I, as given in Supplementary Fig. 6, shows the orientation relationship of [100]_Fe-I_ // [1$\bar 1$0]_FeO_, which is the same as that in Fig. 2. The interface planes were determined to be {010}_Fe-I_ and {110}_FeO_. Therefore, in addition to the topotactic transformation observed through in situ ED measurements, complimentary HAADF imaging enabled direct visualization of the atomic-scale sharp interfaces between the involved phases, and elucidation of their spatial correlation at the reaction front.

### Ionic transport during topotactic reaction

**Fig. 4:**
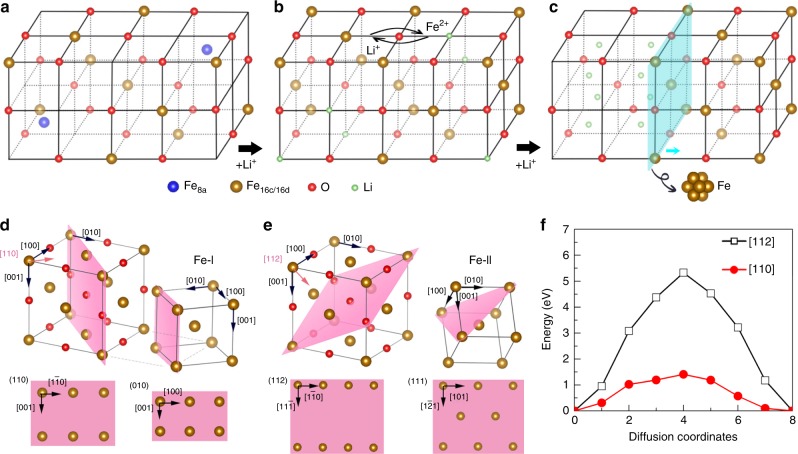
Ionic transport during the topotactic reaction in Fe_3_O_4_. **a**–**c** Schematic illustration of co-operative transport of Li and Fe ions, and consequential atomic re-arrangement within the ccp O-anion framework. The O-framework is retained throughout the entire transformation process, from the initial Fe_3_O_4_, to intermediates of Li_*x*_Fe_3_O_4_, FeO, and final phases of Li_2_O and Fe (further elaborated in Supplementary Fig. 6). During initial lithiation, Li insertion into 16c sites leads to migration of Fe from 8a to 16c sites, turning Fe_3_O_4_ (**a**) to Li_*x*_Fe_3_O_4_ (**b**). Further lithiation causes local segregation of Li and Fe ions (marked by the arrows in (**b**)), and consequently the formation Li_2_O and FeO (left and right, respectively in (**c**)), with an interface between the two (marked by a cyan plane). Continuous lithiation leads to the growth of Li_2_O domains and so the propagation of the interface to the FeO side (marked by a cyan arrow in (**c**)), and concomitantly the nucleation of Fe from the interfacial region. **d**, **e** Atomistic models for the diffusion of Fe ions along two directions, [110] and [112] (indicated by pink arrows), which leads to cationic re-arrangement towards forming Fe-I and Fe-II, respectively within the ccp framework. The interface planes perpendicular to the Fe diffusion directions are marked using pink color. **f** Energy barriers of Fe diffusion along the two directions, namely [110] (red) and [112] (black), computed using the structure models in (**d**) and (**e**), respectively. The calculated atomic structures of the involved intermediates are given in Supplementary Fig. 7

A combination of in situ ED (in the bulk) and HAADF imaging (local), provided details of the reaction process, regarding the phase transformation from Fe_3_O_4_ to Li_*x*_Fe_3_O_4_, to FeO and then to Li_2_O/Fe phases, as schematically illustrated in Fig. 4a–c and Supplementary Fig. 7. In combination with the insight provided by previous work^12^, Fig. 4 also shows how Li and Fe ions are relocated within the ccp framework. Briefly speaking, the process consists of three steps: (i) Relocation of Fe ions with initial lithiation, during which Fe ions move from tetrahedral 8a to octahedral 16c sites as Li ions insert into the 16c sites, leading to the formation of Li_*x*_Fe_3_O_4_ (Fig. 4a, b)^12^. (ii) Local Li/Fe migration with continuous lithiation, during which atomic rearrangement occurs within the ccp framework, leading to the formation of Li_2_O and FeO domains (Fig. 4c)^12^. Since all these phases share the same ccp framework, it only causes small changes in the host structure with small lattice mismatch, i.e., only ~2.5% mismatch along [100] direction between Fe_3_O_4_ and FeO, and ~6% between FeO and Li_2_O. (iii) Fe extrusion with further lithiation, during which Li_2_O grows at the expense of FeO, and concomitantly, nucleation of Fe clusters occurs preferably at the Li_2_O/FeO boundaries, facilitated by the strain-induced space at boundaries (Fig. 4c)^12^. Two types of Fe nanograins (Fe-I and Fe-II; shown in Fig. 2a) possess different orientation relationships with other phases, which should be determined by the different pathways of Fe migration within the ccp framework. As illustrated in Fig. 4d and e, there are two open channels for Fe migration, either along the [110] or [112] direction. When Fe ions move along the [110] direction, the interface plane should be perpendicular to the diffusion direction (Fig. 4d), i.e. (110)_ccp_ or (010)_Fe-I_ (as marked in pink), which is consistent with what was observed in the HAADF image (Fig. 3f). The lattice mismatch at the interface is about 5.3% along the [$1\bar 10$] direction of ccp framework, and about 34% along the [001] direction, using the experimental lattice constants for bulk FeO and Fe of 0.86 nm and 0.288 nm, respectively. When the Fe ions migrate along the [112] direction, Fe-II nanograin is formed, with the interface plane of (112)_ccp_ parallel to the (111)_Fe-II_ plane. The value of lattice mismatch at the interface is the same as that of case-I, about 5.3% along the [$11\bar 1$] direction of ccp framework, and about 34% along the [$1\bar 10$] direction. After establishing a unit cell of Fe phase, the orientation relationship between Fe-I/Fe-II and ccp framework turns out to be the same as that experimentally determined in Fig. 2. Such a large mismatch, of about 34%, might be relieved by forming dislocations on the interface between Fe and ccp framework (Supplementary Fig. 6), which were also commonly observed in other conversion or intercalation electrodes^12,33,35^.

Owing to the much higher energy barrier of Fe diffusion along the [112] direction (Fig. 4f), the Fe-II nanograins should be formed in the later stage of lithiation and less frequently than the Fe-I nanograins. The sluggish Fe diffusion along the [112] direction can be attributed to the fact that the Fe ion has to push its way through a channel formed by Fe ions with a width as small as 3.0 Å, as shown in Supplementary Fig. 8a. At the intermediate state, the Fe ions are pushed apart by the diffused Fe ion with a much greater separation of 4.6 Å. In contrast, Fe diffusion along the [110] direction allows the Fe ion to pass through a relatively wide channel with Fe-Fe distance of 5.2 Å (Supplementary Fig. 8d), which is only slightly enlarged at the intermediate state (5.5 Å). Therefore, the barrier for Fe migration is relatively low, below 1.4 eV (Fig. 4f), similar to the values obtained from experimental measurements^36–38^. Although the calculated barrier for Fe diffusion along the [112] direction is much higher, it should be noted that the existence of nearby Li ions, which was not considered in the calculations, would be a driving force large enough for the Fe ions to overcome the energy barrier and diffuse to the vacancy sites. In addition, the interfacial mismatch between FeO and Li_2_O domains (~6%) may further lower the diffusion barrier and facilitate the formation of Fe-II nanograins. Therefore, it is reasonable that the appearance of Fe-II nanograins was only detected after the formation of FeO domains.

## Discussion

Compared to the transport of Li^+^ ions with an energy barrier lower than 1 eV^39^, the higher energy barrier for Fe^2+/3+^ migration may be due to stronger coulombic interaction with surrounding anions/cations. As also demonstrated in Galvanostatic Intermittent Titration Technique (GITT) type measurements (Supplementary Fig. 9), the kinetics of initial Li intercalation is surprisingly lower than that during conversion in the later stage^12^, which contradicts the traditional view of the slow conversion reaction. The results suggest that the energy barrier for Fe migration, even from 8a to the neighboring 16c, is indeed high. Furthermore, the improved kinetics during conversion indicates that the energy barrier for Li/Fe migration is reduced once nano-sized FeO and Li_2_O domains are formed, as suggested from theoretical calculations^40^. Due to the sluggish movement of Fe ions, some intermediates of Fe_*x*_Li_2−*x*_O may also exist, similar to the observation of Fe_*x*_Li_2−*x*_F_2_ during conversion reaction in iron fluoride^6^.

From the view of structural changes, the topotactic transformation during conversion reaction in Fe_3_O_4_ resembles that of intercalation process, during which the host structure of the ccp framework is maintained despite abrupt phase transformations, going through intermediate phases of completely different structures, such as Li_*x*_Fe_3_O_4_, FeO and Li_2_O^12^. The delithiation process takes place through the reaction: Fe + Li_2_O → FeO + 2Li^+^, during which the ccp framework is still maintained due to the same framework in both of FeO and Li_2_O phases^12^. Therefore, during the MET reaction, the sustenance of the ccp framework during conversion and re-conversion processes, appears to be  cruical to the structural integrity and high reversibility in Fe_3_O_4_. On the other hand, the transformation from Li_*x*_Fe_3_O_4_ to FeO and Li_2_O is sluggish (Fig. 2b), likely due to the slow process of long-range re-ordering and segregation of Fe and Li ions, as suggested in the previous work^12^. It is not electronic transport, but ionic transport involving sluggish migration of Fe ions, that appears to be the main limiting step of the kinetics during MET reaction. Therefore, designing nano-sized Fe_3_O_4_ particles for shortening the path of ionic transport is important to enabling both high-rate capability and cyclability, as demonstrated in nano-sized Fe_3_O_4_ (Supplementary Fig. 10), and  previous observation in the nano-architectured Fe_3_O_4_^22^.

The low energy efficiency of conversion electrodes, another major concern rooted in the large voltage hysteresis during cycling, has been demonstrated in Fe_3_O_4_ despite its high-rate capability in the nano-architectured electrodes^22^. A detailed theoretical study has demonstrated that the voltage hysteresis in conversion electrodes arises from two thermodynamic factors: (1) a large difference in the diffusivity between Li and TM ions and (2) an insufficient thermodynamic driving force for the TM ions to re-enter the structure during lithiation^41^, which should also be applicable to Fe_3_O_4_. Due to the sluggish migration of Fe ions with a larger energy barrier of ~1.4 eV than that of Li ions (below 1 eV), the Li-ion migration only occurs after Fe ions move out to make room for Li ions during lithiation. But Li ions can first move out of the structure through many open channels during delithiation, and thus are not limited by Fe insertion. Such different reaction pathways during lithiation and delithiation may be one origin of voltage hysteresis. But it may only cause a small degree of hysteresis, because we should note that Li_2_O and FeO phases have similar structure with the same ccp framework, similar as the situation in Li_3_Sb^42^. Therefore, we expect that the main part of hysteresis results from the driving force for Fe extrusion during lithiation and Fe insertion during delithiation^42^. Further studies on the evolution of Fe chemical potential during lithiation and delithiation are needed for elucidating and reducing the hysteresis in Fe_3_O_4_; but findings from studies on metal fluorides, particularly on the kinetic nature of the voltage hysteresis and its reduction via tuning ionic transport by cationic substitution may also be applied to Fe_3_O_4_, indicating the potential of making it a viable electrode in commercial batteries^43,44^.

### Conclusion

In summary, we investigated the dynamic MET reaction process in Fe_3_O_4_ through time-resolved in situ ED and spatially-resolved atomic imaging. The use of single crystal in this study enabled us to reveal the crystallographic correlation among all the involved phases, and the co-operative migration of Li and Fe ions within the ccp O-anion framework. In contrast to conventional view of dramatic collapse of the host structure during conversion, this study provided direct evidences of a topotactic transformation with the ccp oxygen framework retained, thereby enabling structural integrity and high cyclability in Fe_3_O_4_. Ionic transport involving sluggish Fe^2+/3+^ migration in Fe_3_O_4_ was elucidated through first-principles calculations, and enhancing rate capability and high cyclability by nanosizing was also demonstrated. The findings may help to pave the way for designing viable MET electrode materials for next-generation high energy LIBs.

The developed platform for in situ single-crystal electron diffraction may be applied broadly to studies of electrochemical reaction in various electrode materials, to reveal crystallographic correlation among the intermediates at the reaction front, which is typically obfuscated by random orientation of constituent crystallites in the commonly employed polycrystalline samples.

## Supplementary information

Supplementary Information

Supplementary Movie 1

Supplementary Movie 2

## Data Availability

All data generated or analyzed during this study are included in this published article and its supplementary information files.

## References

[1] Whittingham MS (2014). Ultimate limits to intercalation reactions for lithium batteries. Chem. Rev..

[2] Kraytsberg A, Ein-Eli Y (2017). A critical review-promises and barriers of conversion electrodes for Li-ion batteries. J. Solid State Electrochem..

[3] Yu SH, Feng X, Zhang N, Seok J, Abruña HD (2018). Understanding conversion-type electrodes for lithium rechargeable batteries. Acc. Chem. Res..

[4] Lou XW, Deng D, Lee JY, Feng J, Archer LA (2008). Self-supported formation of needlelike Co_3_O_4_ nanotubes and their application as lithium-ion battery electrodes. Adv. Mater..

[5] Yan C (2018). Heterogeneous molten salt design strategy toward coupling cobalt–cobalt oxide and carbon for efficient energy conversion and storage. Adv. Energy Mater..

[6] Ko JK (2014). Transport, phase reactions, and hysteresis of iron fluoride and oxyfluoride conversion electrode materials for lithium batteries. ACS Appl. Mater. Interfaces.

[7] Cabana J, Monconduit L, Larcher D, Palacin MR (2010). Beyond intercalation-based Li-ion batteries: the state of the art and challenges of electrode materials reacting through conversion reactions. Adv. Mater..

[8] Poizot P, Laruelle S, Grugeon S, Dupont L, Tarascon J (2000). Nano-sized transition-metal oxides as negative-electrode materials for lithium-ion batteries. Nature.

[9] Amatucci GG, Pereira N (2007). Fluoride based electrode materials for advanced energy storage devices. J. Fluor. Chem..

[10] Wang F (2011). Conversion reaction mechanisms in lithium ion batteries: study of the binary metal fluoride electrodes. J. Am. Chem. Soc..

[11] Wang F (2012). Tracking lithium transport and electrochemical reactions in nanoparticles. Nat. Commun..

[12] Zhang W (2016). Insights into ionic transport and structural changes in magnetite during multiple-electron transfer reactions. Adv. Energy Mater..

[13] McKenna KP (2014). Atomic-scale structure and properties of highly stable antiphase boundary defects in Fe_3_O_4_. Nat. Commun..

[14] Ye XR, Daraio C, Wang C, Talbot J, Jin S (2006). Room temperature solvent-free synthesis of monodisperse magnetite nanocrystals. J. Nanosci. Nanotechnol..

[15] Bock DC (2015). 2D cross sectional analysis and associated electrochemistry of composite electrodes containing dispersed agglomerates of nanocrystalline magnetite, Fe_3_O_4_. ACS Appl. Mater. Interfaces.

[16] Bock DC (2017). Size dependent behavior of Fe_3_O_4_ crystals during electrochemical (de) lithiation: an in situ X-ray diffraction, ex situ X-ray absorption spectroscopy, transmission electron microscopy and theoretical investigation. Phys. Chem. Chem. Phys..

[17] Bock DC (2016). Dispersion of nanocrystalline Fe_3_O_4_ within composite electrodes: Insights on battery-related electrochemistry. ACS Appl. Mater. Interfaces.

[18] Bruck AM (2016). Nanocrystalline iron oxide based electroactive materials in lithium ion batteries: the critical role of crystallite size, morphology, and electrode heterostructure on battery relevant electrochemistry. Inorg. Chem. Front.

[19] Menard MC, Takeuchi KJ, Marschilok AC, Takeuchi ES (2013). Electrochemical discharge of nanocrystalline magnetite: structure analysis using X-ray diffraction and X-ray absorption spectroscopy. Phys. Chem. Chem. Phys..

[20] Bock DC, Marschilok AC, Takeuchi KJ, Takeuchi ES (2017). Deliberate modification of the solid electrolyte interphase (SEI) during lithiation of magnetite, Fe_3_O_4_: impact on electrochemistry. Chem. Commun..

[21] Mitra S, Poizot P, Finke A, Tarascon JM (2006). Growth and electrochemical characterization versus lithium of Fe_3_O_4_ electrodes made by electrodeposition. Adv. Funct. Mater..

[22] Taberna PL, Mitra S, Poizot P, Simon P, Tarascon JM (2006). High rate capabilities Fe_3_O_4_-based Cu nano-architectured electrodes for lithium-ion battery applications. Nat. Mater..

[23] Xu X (2018). Facile synthesis of three-dimensional Cu/Fe_3_O_4_ nanowires as binder-free anode for lithium-ion batteries. Appl. Surf. Sci..

[24] Abraham A (2016). Investigating the complex chemistry of functional energy storage systems: the need for an integrative, multiscale (molecular to mesoscale) perspective. ACS Cent. Sci..

[25] Knehr KW (2017). Simulations of lithium-magnetite electrodes incorporating phase change. Electrochim. Acta.

[26] Lininger CN, Brady NW, West AC (2018). Equilibria and rate phenomena from atomistic to mesoscale: simulation studies of magnetite. Acc. Chem. Res..

[27] Yao Z (2017). Revealing the conversion mechanism of transition metal oxide electrodes during lithiation from first-principles. Chem. Mater..

[28] Badway F, Cosandey F, Pereira N, Amatucci G (2003). Carbon metal fluoride nanocomposites high-capacity reversible metal fluoride conversion materials as rechargeable positive electrodes for Li batteries. J. Electrochem. Soc..

[29] Wang H, Wang F (2016). In situ, operando measurements of rechargeable batteries. Curr. Opi. Chem. Eng..

[30] He K (2016). Visualizing non-equilibrium lithiation of spinel oxide via in situ transmission electron microscopy. Nat. Commun..

[31] Huang JY (2010). In situ observation of the electrochemical lithiation of a single SnO_2_ nanowire electrode. Science.

[32] Zhang W (2018). Localized concentration reversal of lithium during intercalation into nanoparticles. Sci. Adv..

[33] Zhu Y (2013). In situ atomic-scale imaging of phase boundary migration in FePO_4_ microparticles during electrochemical lithiation. Adv. Mater..

[34] Thackeray M, David W, Goodenough J (1982). Structural characterization of the lithiated iron oxides Li_x_Fe_3_O_4_ and Li_x_Fe_2_O_3_ (0<x<2). Mater. Res. Bull..

[35] Ulvestad A (2015). Topological defect dynamics in operando battery nanoparticles. Science.

[36] Wuensch B, Vasilos T (1962). Diffusion of transition metal ions in single‐crystal MgO. J. Chem. Phys..

[37] Carter R, Richardson F (1954). An examination of the decrease of surface-activity method of measuring self-diffusion coefficients in wustite and cobaltous oxide. JOM.

[38] Himmel L, Mehl R, Birchenall CE (1953). Self-diffusion of iron in iron oxides and the Wagner theory of oxidation. JOM.

[39] Islam M, Catlow C (1988). Lithium insertion into Fe_3_O_4_. J. Solid State Chem..

[40] Tibbetts K, Miranda CR, Meng YS, Ceder G (2007). An ab initio study of lithium diffusion in titanium disulfide nanotubes. Chem. Mater..

[41] Yu HC (2014). Designing the next generation high capacity battery electrodes. Energ. Environ. Sci..

[42] Chang D, Chen MH, Van der Ven A (2015). Factors contributing to path hysteresis of displacement and conversion reactions in Li ion batteries. Chem. Mater..

[43] Li L (2016). Origins of large voltage hysteresis in high-energy-density metal fluoride lithium-ion battery conversion electrodes. J. Am. Chem. Soc..

[44] Wang F (2015). Ternary metal fluorides as high-energy cathodes with low cycling hysteresis. Nat. Commun..

