# Supplementary material for: Multi-electron transfer enabled by topotactic reaction in magnetite
Source: Nat Commun. 2019 Apr 29;10:1972. doi: 10.1038/s41467-019-09528-9 (PMC6488677; doi:10.1038/s41467-019-09528-9)
Supplement: Supplementary file 1 — Supplementary Information [file 41467_2019_9528_MOESM1_ESM.pdf]

Supplementary Information

Multi-Electron Transfer Enabled by Topotactic Reaction in Magnetite

Zhang et al.

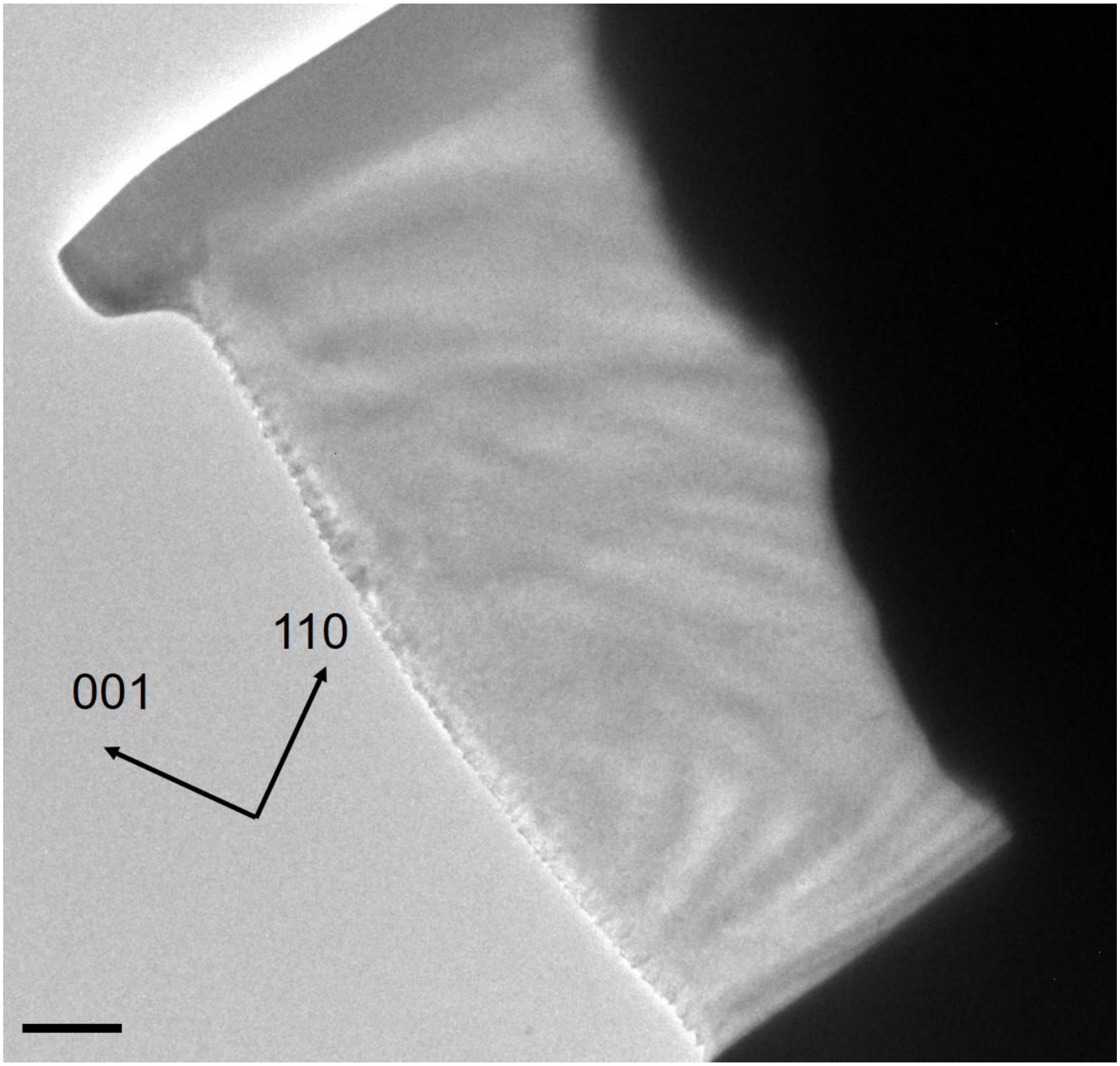

Supplementary Figure 1 A TEM image of a single-crystal Fe_3O_4 projected along [1-10] direction. The sample was used to obtain the time-resolved diffraction patterns in Supplementary Figure 4. Scale bar: 500 nm.

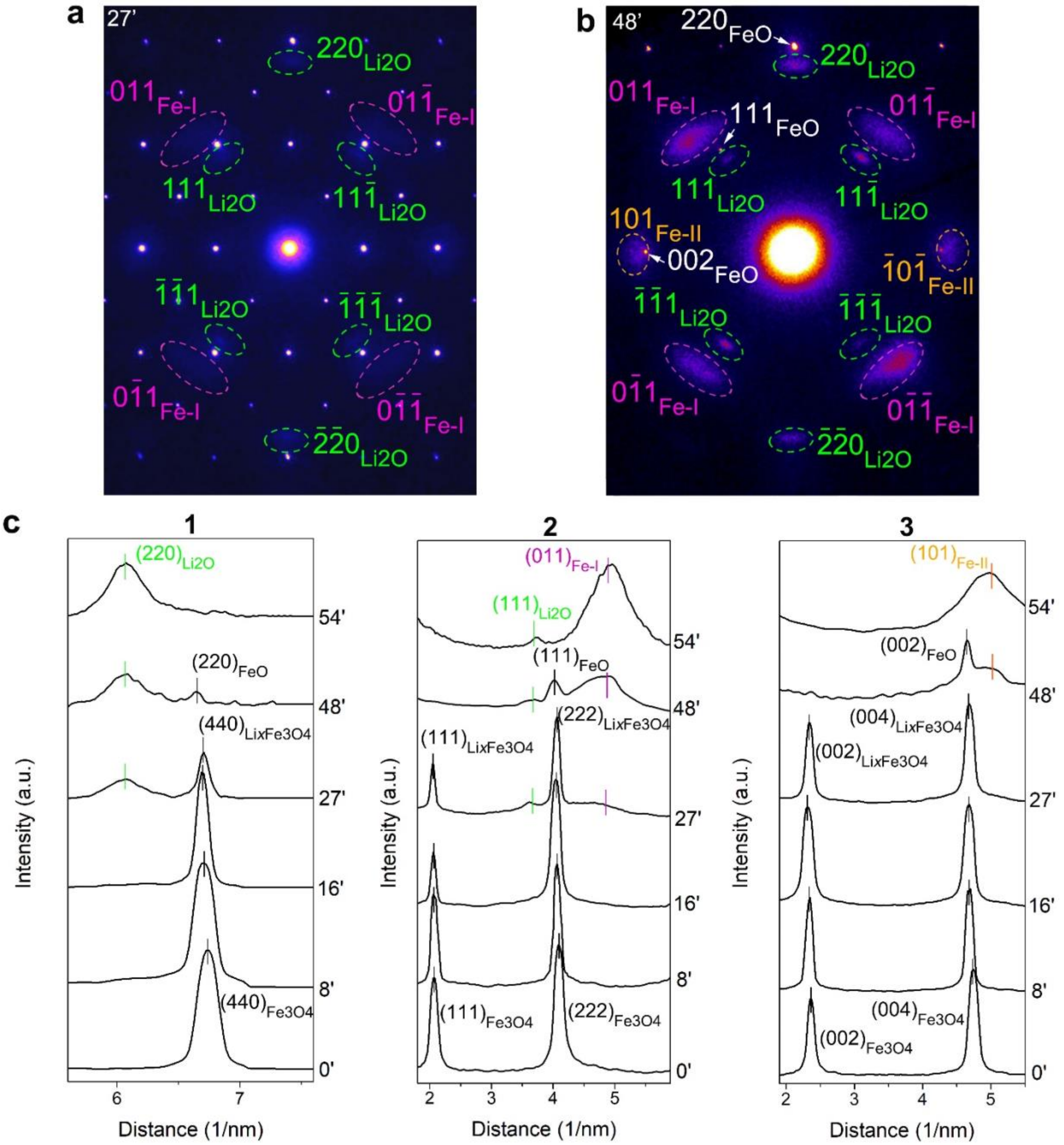

Supplementary Figure 2 Topotactic transformation during conversion reaction in a single-crystal Fe_3O_4 revealed by *in situ* electron diffraction. (a-b) Time-resolved diffraction patterns at 27 and 48 minutes, respectively. The patterns were obtained from the same sample used to generate the data for Figure 2. Different diffraction spots of Li_2O, Fe-I and Fe-II were marked by green, purple and orange circles, respectively, while the spots of FeO phase were marked by white arrows. The sharp white spots in (a) are related to the $Li_xFe_3O_4$ phase. (c) Evolution of intensity profiles obtained from line scan along 1, 2 and 3 in Fig. 2a.

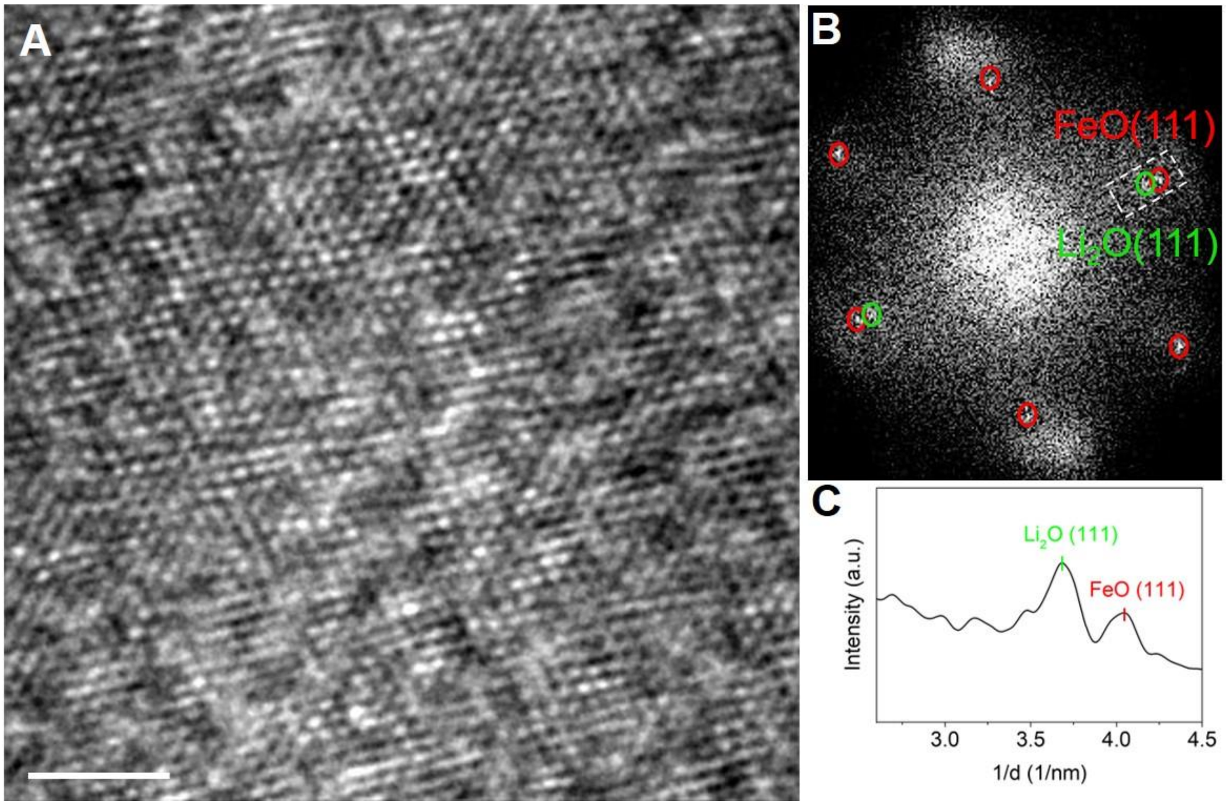

Supplementary Figure 3 Highly structurally integrated FeO and Li_2O domains in partially lithiated regions. (A) HRTEM image of a partially lithiated region. Scale bar: 2 nm. (B) FFT pattern taken from the whole region in (B), demonstrating the coherent connection between FeO and Li_2O domains in (B). (C) Intensity profile obtained from the line scan across the region marked by white box in (B), showing the existence of $(111)_{Li2O}$ and $(111)_{FeO}$ peaks. According to the FFT pattern in (B), we identified the crystallographic relationship between FeO and Li_2O in this local region is in the following: $[1\bar{1}0]_{FeO}$ // $[1\bar{1}0]_{Li2O}$, $(111)_{FeO}$ // $(111)_{Li2O}$. It is exactly the same as that observed by *in situ* electron diffraction (Fig. 2a and b).

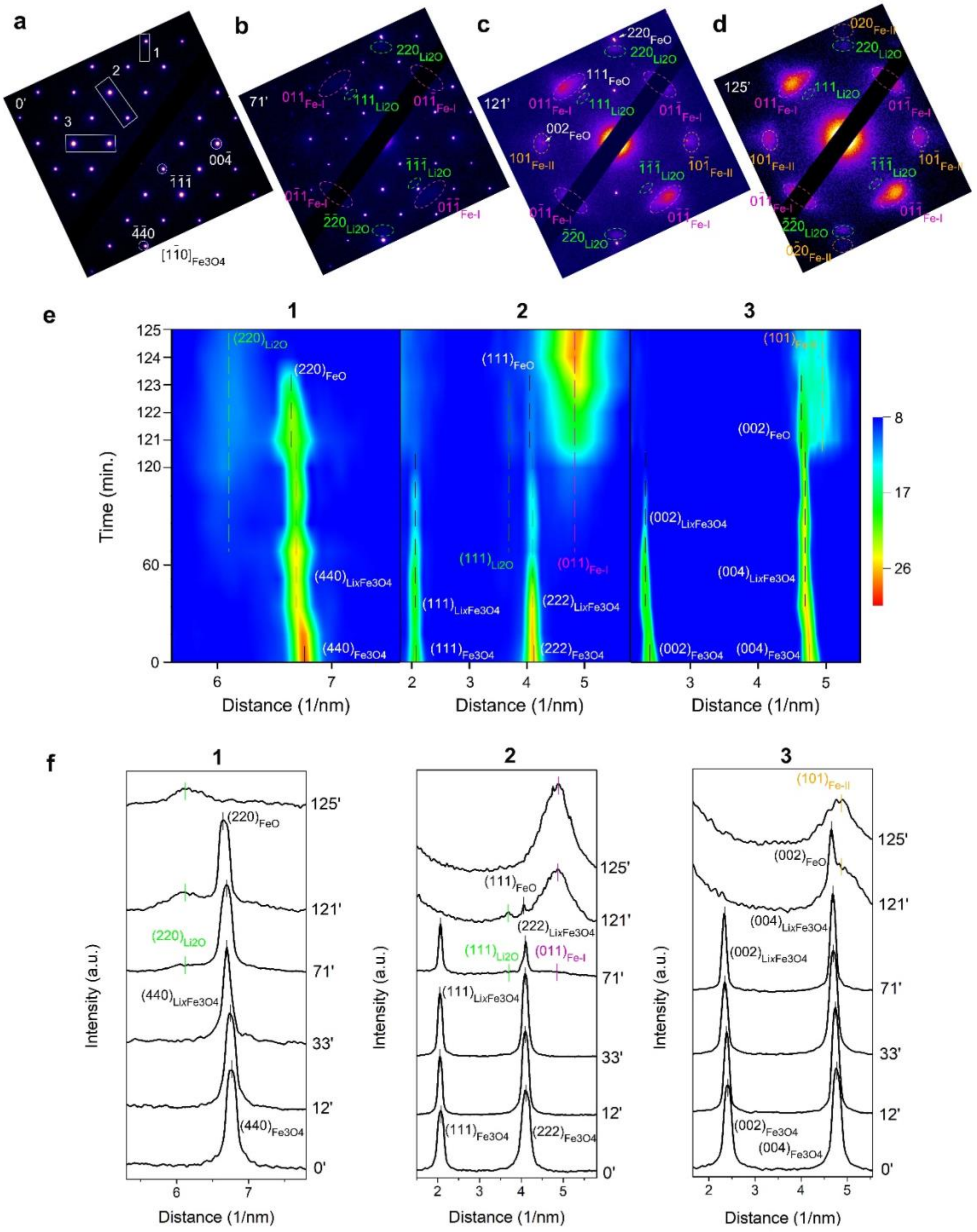

Supplementary Figure 4 Topotactic transformation during conversion reaction in another single-crystal Fe_3O_4 (Supplementary Figure 1) revealed by *in situ* electron diffraction. (a-d) Time-resolved diffraction patterns at different lithiation states. The sharp white spots in (a) and (b) are related to the Fe_3O_4 and $Li_xFe_3O_4$ phases, respectively. (e) Color map of intensity profiles obtained from line scan along 1, 2 and 3 in (a). (f) Evolution of intensity profiles at different lithiation states, corresponding to the color map in (e).

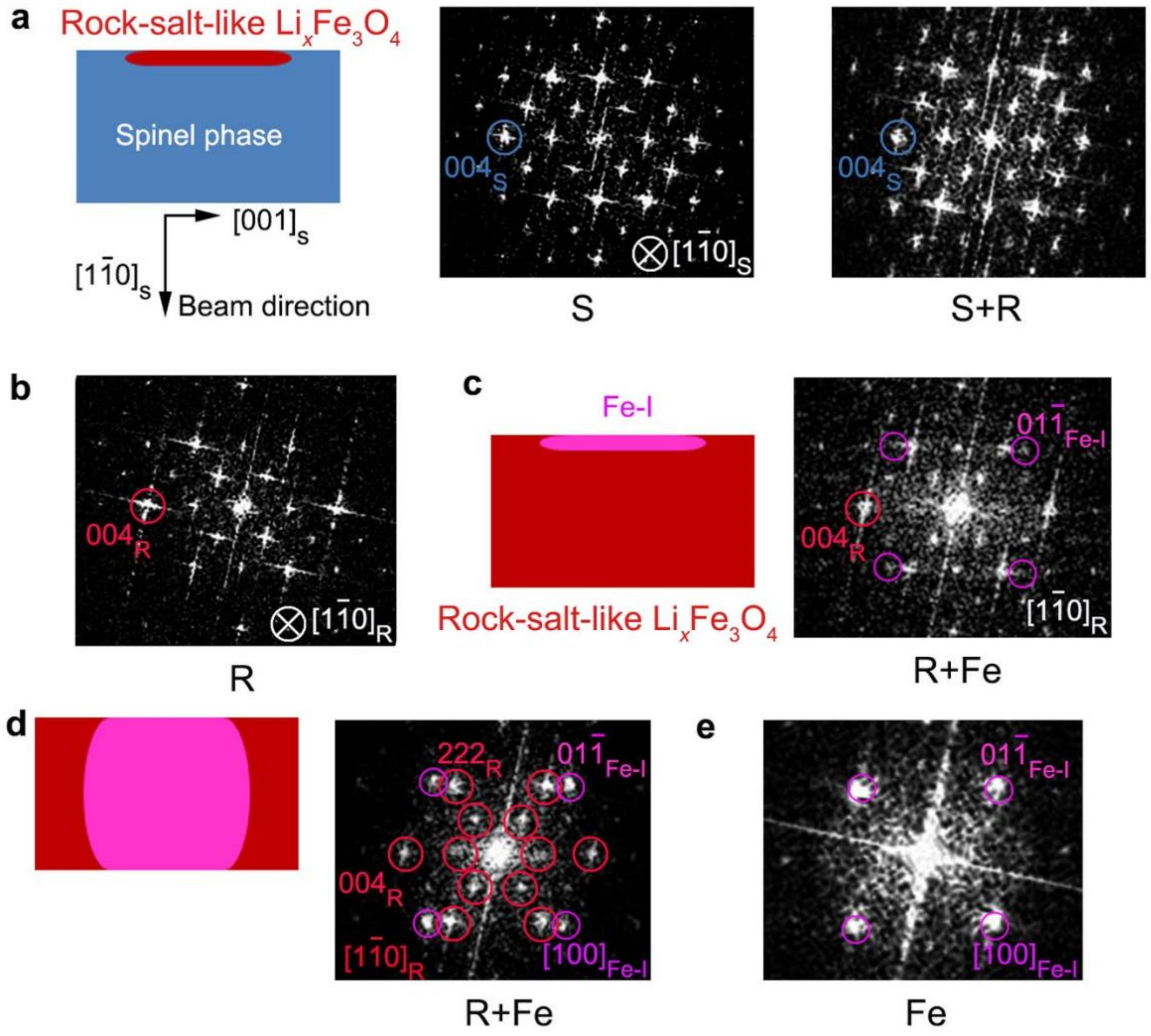

Supplementary Figure 5 ***Fast Fourier transform*** **(*FFT*) patterns corresponding to the HAADF images in Figure 3.** (a) Schematic illustration showing the formation of rock-salt-like $Li_xFe_3O_4$ phase (R, red color) on the surface of pristine spinel Fe_3O_4 phase (S, blue color). Owing to the projection view of HAADF image along the [1-10] direction, the central region is a mixture of $Li_xFe_3O_4$ and Fe_3O_4 phases, as shown in the region marked by white dashed line in Fig. 3b. The insets are the FFT patterns of Fe_3O_4 and a mixture of $Li_xFe_3O_4$ and Fe_3O_4, which are obtained from the corresponding HAADF image in Fig. 3b. (b) FFT pattern of rock-salt-like $Li_xFe_3O_4$ phase obtained from the HAADF image of $Li_xFe_3O_4$ phase in Fig. 3c. The pattern is the same as that in Supplementary Figure 2a and 4b. (c) Schematic illustration showing the formation of Fe-I nanoparticles (purple color) on the surface of rock-salt-like $Li_xFe_3O_4$ phase (red color). The spots of Fe-I particles were marked by purple circles in the inset FFT pattern. The FFT pattern corresponds to the HAADF image in Fig. 3d. (d) Schematic illustration showing the growth of Fe-I nanoparticles inside the rock-salt-like $Li_xFe_3O_4$ phase. Owing to the growth of Fe-I particles, the intensity of diffraction spots of Fe-I particles increased, comparing to that in (c). The FFT pattern was obtained from the HAADF image in Fig. 3e. (e) FFT pattern of pure Fe-I nanoparticle obtained from Fig. 3f.

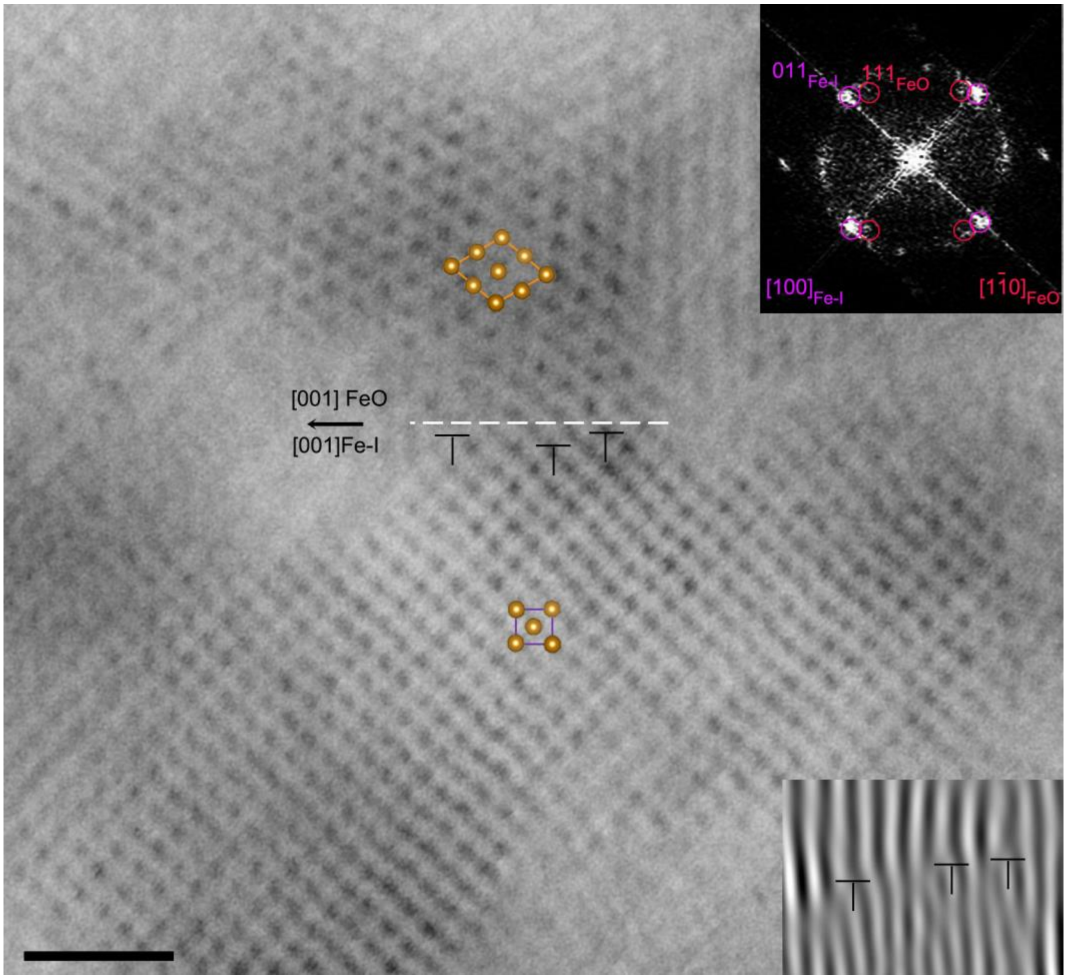

Supplementary Figure 6 ABF image showing the structural correlation between FeO and Fe-I phases. The corresponding FFT pattern in the upper-right corner shows the orientation relationship between FeO and Fe-I phases, which is $[100]_{Fe\text{-}I}$ // $[1\text{-}10]_{FeO}$, the same as that observed in *in situ* electron diffraction measurements in Fig. 2 and Supplementary Figure 4. The FFT pattern of FeO phase is the same as that in Supplementary Figure 2b and 4c. The white dashed line marks the interface between FeO and Fe-I phases, corresponding to the $\{010\}_{Fe\text{-}I}$ plane. The interface between Fe-I and FeO is parallel to the direction of [001]Fe-I and [001]FeO. The inset in the lower-right corner is the inverse FFT image corresponding to the local area showing the position of misfit dislocations at the interface between Fe-I and FeO, as marked by ⊤ in the HAADF image. Scale bar: 1 nm.

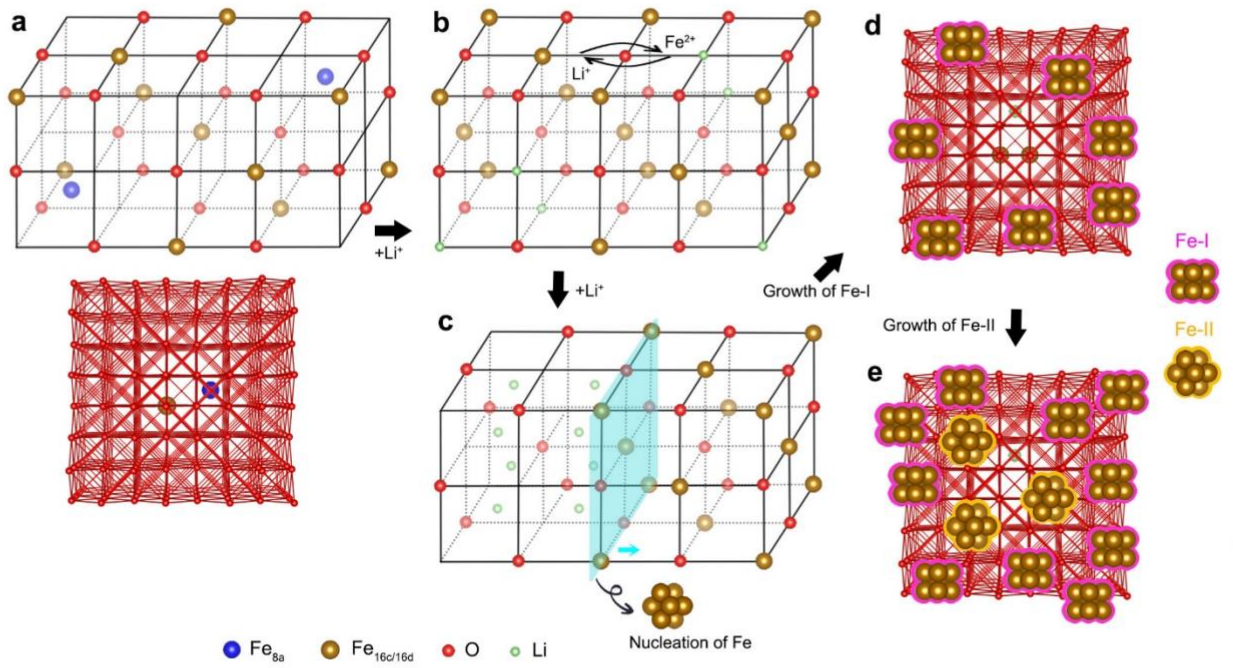

Supplementary Figure 7 Schematic illustration of the topotactic transformation in the ccp O-anion framework. (a) Pristine structure of Fe_3O_4 with Fe occupying 8a (blue) and 16d (brown) sites. The ccp framework is shown by the red mesh. (b) Lithiation inducing the formation of $Li_xFe_3O_4$, with all Li and Fe ions at 16c/16d sites. Local atomic arrangement of Fe and Li ions in (b) leads to the formation of FeO and Li_2O domains in (c). All the Fe_3O_4, $Li_xFe_3O_4$, FeO and Li_2O phases share the same *ccp* framework in (a). Further lithiation in (c) causes the nucleation of Fe clusters at the domain boundaries marked by cyan color. Continuous lithiation causes the growth of Fe-I nanograins within the ccp framework in (d), and then Fe-II nanograins are formed in the rest part of the framework in (e).

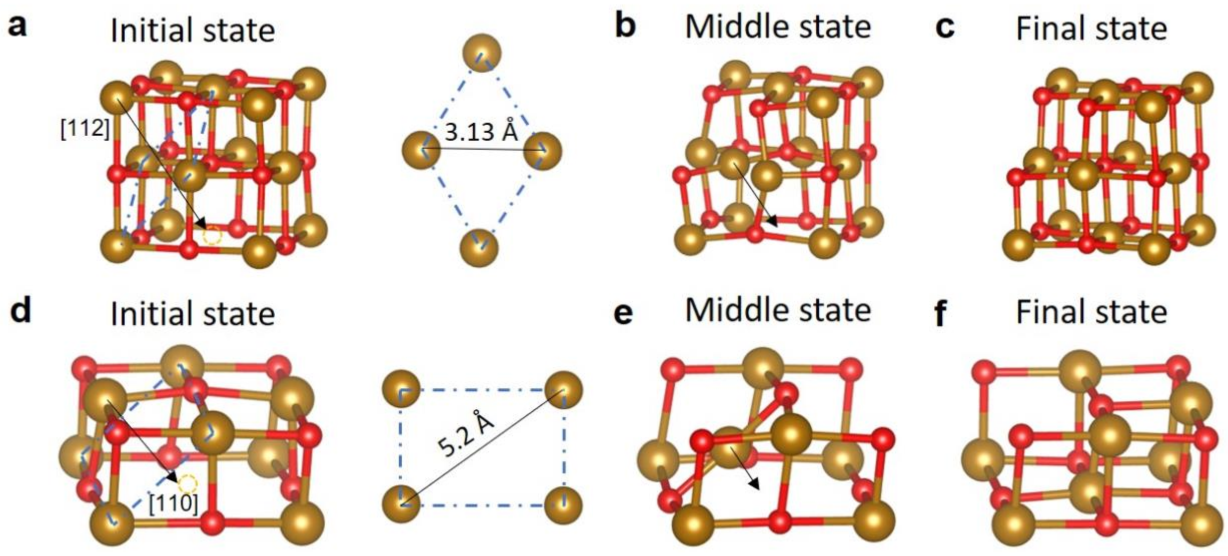

Supplementary Figure 8 Computed atomic arragement of intermediates for Fe diffusion along [112] and [110] directions. (a-c) Atomic structure of initial, middle and final states of Fe diffusion along the [112] direction. (d-f) Atomic structure of initial, middle and final states of Fe diffusion along the [110] direction. The dashed orange circles in (a) and (d) show the Fe vacancies, to which the Fe ions will diffuse. The black arrows in (a) and (d) indicate the diffusion directions. The dashed blue boxes in (a) and (d) show the four-coordinated Fe face, through which the Fe ions will pass in the middle state.

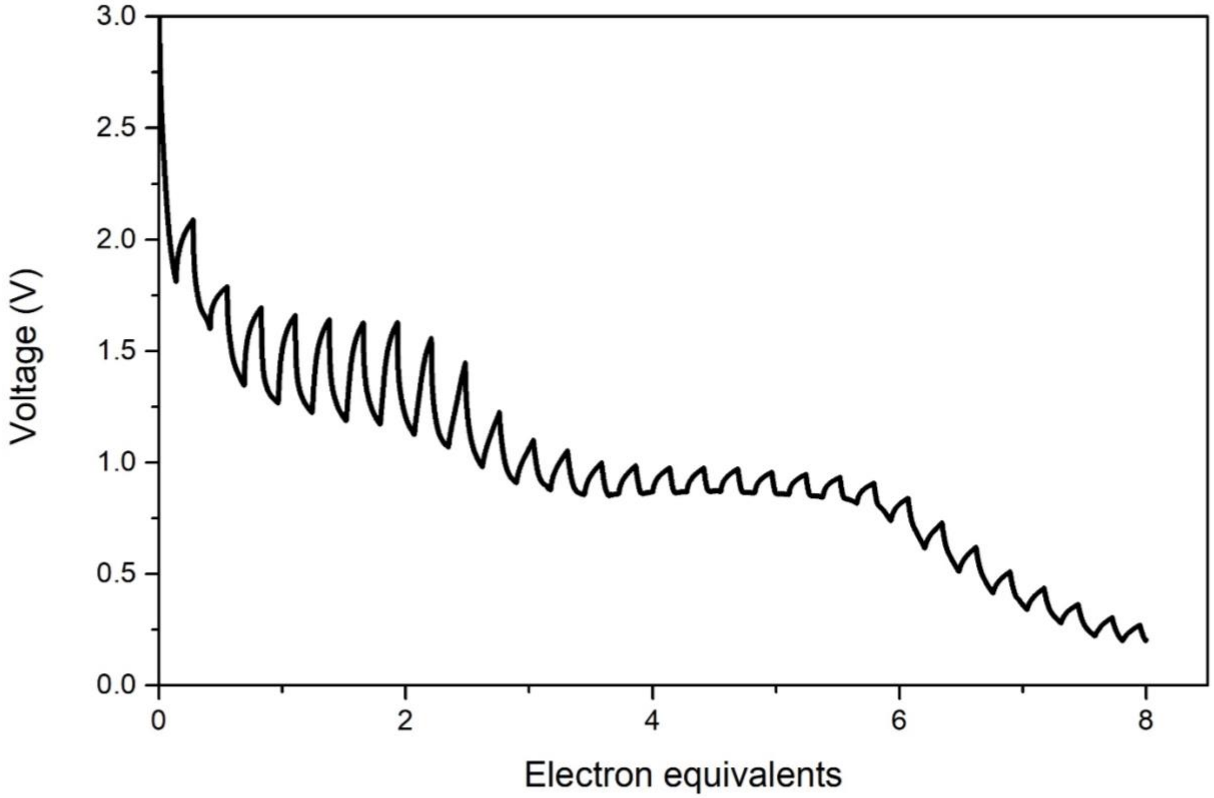

Supplementary Figure 9 Voltage profile from galvanostatic intermittent titration technique (GITT) measurements (at 1/80 C for 5 h followed by a 5 h rest). The polarization is large during the intercalation process above 1.0 V, indicating a sluggish reaction process. The polarization is much reduced during the following conversion process (*i.e.* long plateau below 0.9 V).

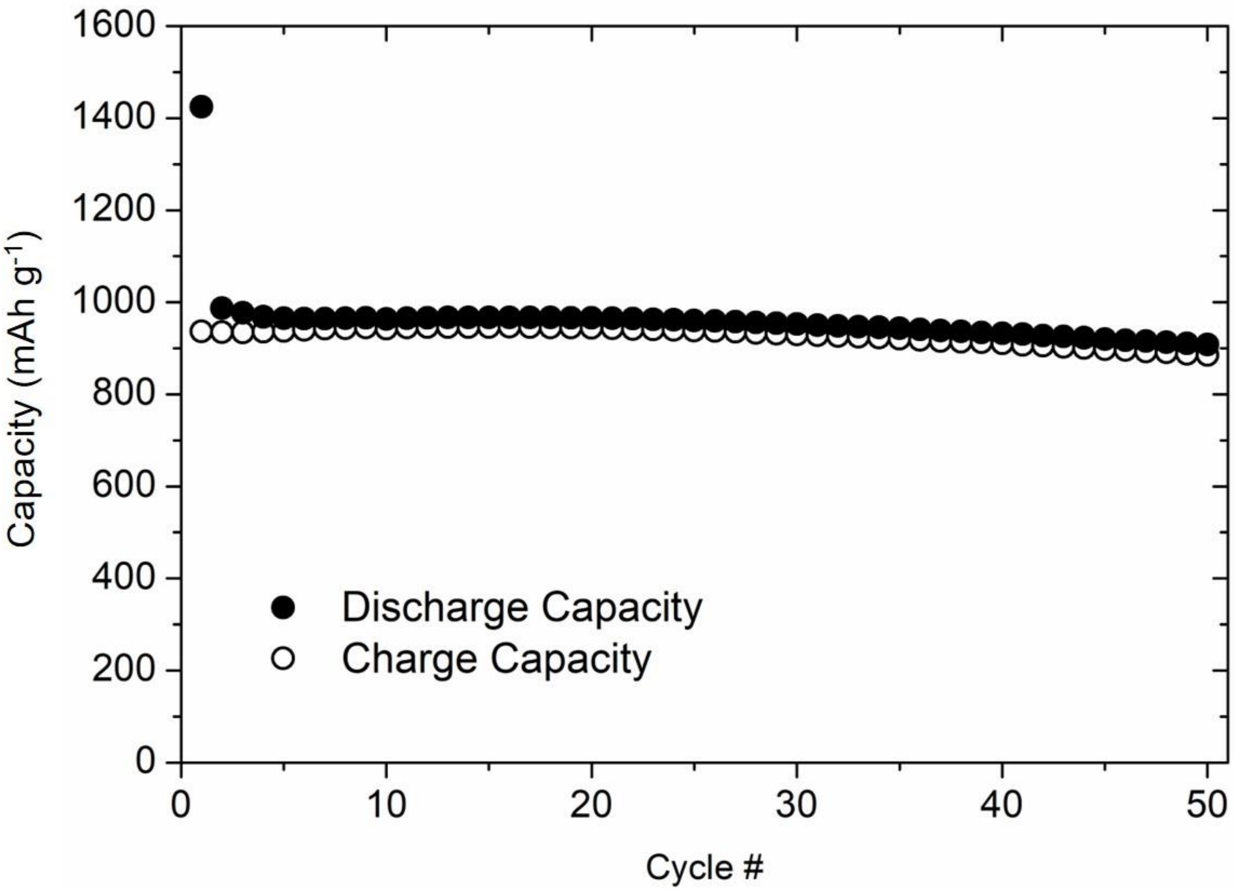

Supplementary Figure 10 Discharge and charge capacities versus cycle number for the Li/Fe_3O_4 coin cells. After first discharge, the coin cells show sustained discharge and charge capacities, with losses in cycles 2 - 50 of 8.1% and 5.3%, respectively.

Supplementary Note 1 *In situ* electron diffraction on a second single crystal The phenomenon of topotactic transformation is repeatable during conversion reaction in the single-crystal Fe_3O_4, as evidenced by another set of time-resolved electron diffraction patterns in Supplementary Figure 4. A similar *in situ* electron diffraction measurement was performed on another single-crystal Fe_3O sample as shown in Supplementary Figure 1. The sample was projected along the [1-10] direction, as shown in Fig.1. After lithiation started, all the peaks of Fe_3O_4 shifted to left until 33 min, indicating the transformation from Fe_3O_4 to $Li_xFe_3O_4$ (Supplementary Figure 4e and f). At 71 min, a portion of $Li_xFe_3O_4$ was fully converted to Li_2O and Fe-I, as evidenced by the appearance of spots of $(220)_{Li2O}$ in 1, $(111)_{Li2O}$ and $(011)_{Fe\text{-}I}$ in 2 (Supplementary Figure 4b, e and f). The rest of $Li_xFe_3O_4$ was fully converted to FeO at 121 min, as indicated by the observation that the peaks of $(111)_{LixFe3O4}$ in 2 and $(002)_{LixFe3O4}$ in 3 disappeared, meanwhile the peaks of $(440)_{LixFe3O4}$, $(222)_{LixFe3O4}$ and $(004)_{LixFe3O4}$ further shifted left and changed to $(220)_{FeO}$, $(111)_{FeO}$ and $(002)_{FeO}$ (Supplementary Figure 4c, e and f). After 121 min, the Fe-II particles started to form, as indicated by the appearance of $(101)_{Fe\text{-}II}$ peak in 3 (Supplementary Figure 4e and f). All the processes are similar to those observed in Fig. 2, confirming the topotactic transformation and retention of ccp framework during conversion reaction in Fe_3O_4.

Supplementary Methods

Sample preparation: TEM samples of Fe_3O_4 single crystals were prepared from a commercial Fe_3O_4 single crystals from SurfaceNet GmbH, using focused ion beam (FIB). The TEM lamelae were produced using the standard in situ lift-out procedure on the Helios 600 Nanolab dual beam FIB with final thinning performed at 5 keV. The thickness of the prepared TEM samples is approximately 50 nm.

Electrochemical tests: Electrodes for coin cells were fabricated by mixing nano-sized Fe_3O_4 powders (~35 nm), Super P carbon, and polyvinylidene fluoride (7:2:1 ratio). Coin cells were constructed using lithium-metal anodes and an electrolyte (1M $LiPF_6$ in 3:7 volume ratio of fluoroethylene carbonate to dimethyl carbonate). The coin cells were cycled between 3.0 and 0.1 V vs. Li/Li^+ at a rate of C/2. Galvanostatic Intermittent Titration Technique (GITT) type measurements were performed by applying an intermittent current for 5 h followed by a 5-h relaxation period at a rate of C/80.

***In situ* experiments and structural characterization:** For the *in situ* electron diffraction (ED) experiments, an *in situ* cell as described in Supplementary Reference 1 was adapted for *in situ* measurements on single crystals. Briefly, the TEM sample prepared by FIB was loaded in a Nanofactory TEM-STM specimen holder. A tungsten tip was used to scratch fresh lithium metal from a cut lithium metal surface in an argon glove box, and then loaded onto the holder. All the components were sealed in an Argon glove box and transferred into the TEM column using an Ar-filled bag. As shown in Fig.1a, the biasing probe touched the sample, and a constant negative bias versus TEM half-grid was applied to initiate the reaction. No reaction or current flow occurred before applying the bias. The battery consists of a TEM grid (current collector), Fe_3O_4 (cathode), and Li metal (anode). A thin passivation layer of LiO_xN_y on the surface of the Li metal acted as the solid electrolyte.

The *in situ* ED patterns were recorded in a JEOL 2100F microscope at 200 KV with a 0.23 nm point-to-point spatial resolution. The TEM and HAADF images were recorded from the lithiated samples using a JEOL ARM 200F microscope equipped with two aberration correctors and a cold-field-emission electron source. The electron-beam damage was minimized by spreading the electron beam to the largest beam diameter, to make sure the *in situ* ED measurement reveals the state of phase transformation without artifacts.

***Ab initio* calculations:** Spin-polarized DFT+U calculations were carried out with the projected augmented wave method implemented in the Vienna *ab initio* simulation package (VASP) [2], using the PBE exchange-correlation functional and a kinetic energy cutoff of 400 eV. A Hubbard U correction of Ueff= 5.3 eV was applied to the Fe *d*-orbitals. A cubic 2 × 2 × 2 supercell was used to model the Rocksalt structure of FeO with the type-II antiferromagnetic ordering, i.e. with stacking of alternating ferromagnetic planes in the [111] direction, and an experimental lattice constant of 8.666 Å was adopted. The small rhombohedral or tetragonal distortions are neglected. The magnetic moment of Fe was computed to be 3.7 μB, which is consistent with previous DFT+U studies [3]. We used the nudged elastic-band method [4] to calculate migration pathways and estimated the activation energy for the diffusion of a single Fe vacancy (at the 16c site) along the [110] and [112] directions, respectively.

Supplementary References

1. Wang, F. *et al.* Tracking lithium transport and electrochemical reactions in nanoparticles. *Nat. Commun.* **3**, 1201 (2012).
2. G. Kresse, J. Hafner, Ab initio molecular-dynamics simulation of the liquid-metal–amorphous-semiconductor transition in germanium. *Phys. Rev. B* **49**, 14251 (1994)
3. C. Rödl, F. Fuchs, J. Furthmüller, and F. Bechsted, Quasiparticle band structures of the antiferromagnetic transition-metal oxides MnO, FeO, CoO, and NiO. *Phys. Rev. B* **79**, 235114 (2009).
4. G. Henkelman, B.P. Uberuaga, and H. Jónsson, A climbing image nudged elastic band method for finding saddle points and minimum energy paths. *J. Chem. Phys*. **113**, 9901–9904 (2000).
